# Analgesia in pediatric trauma patients in physician-staffed Austrian helicopter rescue: a 12-year registry analysis

**DOI:** 10.1186/s13049-021-00978-z

**Published:** 2021-11-18

**Authors:** Christopher Rugg, Simon Woyke, Julia Ausserer, Wolfgang Voelckel, Peter Paal, Mathias Ströhle

**Affiliations:** 1 Department of Anaesthesiology and Critical Care Medicine, Medical University of Innsbruck, Anichstrasse 35, 6020 Innsbruck, Austria; 2 Department of Anaesthesiology and Intensive Care Medicine AUVA Trauma Centre Salzburg, Academic Teaching Hospital of the Paracelsus Medical University, Dr.-Franz-Rehrl-Platz 5, 5010 Salzburg, Austria; 3 Christophorus Flugrettungsverein, Baumgasse 129, 1030 Vienna, Austria; 4 Network for Medical Science, University of Stavanger, Stavanger, Norway; 5 Department of Anaesthesiology and Intensive Care Medicine, Hospitallers Brothers Hospital, Paracelsus Medical University, Kajetanerplatz 1, 5010 Salzburg, Austria; 6 Austrian Society for Mountain and High-Altitude Medicine (ÖGAHM), Lehnrain 30a, 6414 Mieming, Austria

**Keywords:** Children, Air ambulance, Analgesia, Accident, Emergency medicine services, Wounds and injuries

## Abstract

**Background:**

As pediatric patients are typically rare among helicopter emergency medical systems (HEMS), children might be at risk for oligo-analgesia due to the rescuer’s lack of experience and the fear of side effects.

**Methods:**

In this retrospective analysis, data was obtained from the ÖAMTC HEMS digital database including 14 physician staffed helicopter bases in Austria over a 12-year timeframe. Primary missions involving pediatric trauma patients (< 15 years) not mechanically ventilated on-site were included. Analgesia was assessed and compared between the age groups 0–5, 6–10 and 11–14 years.

**Results:**

Of all flight missions, 8.2% were dedicated to children < 15 years. Analgetic drugs were administered in 31.4% of all primary missions (3874 of 12,324), wherefrom 2885 were injured and non-ventilated (0–5 yrs.: n = 443; 6–10 yrs.: n = 902; 11–14 yrs.: n = 1540). The majority of these patients (> 75%) suffered moderate to severe pain, justifying immediate analgesia. HEMS physicians typically chose a monotherapy with an opioid (n = 1277; 44.3%) or Esketamine (n = 1187; 41.1%) followed by the combination of both (n = 324; 11.2%). Opioid use increased (37.2% to 63.4%) and Esketamine use decreased (66.1% to 48.3%) in children < 6 vs. > 10 years. Esketamine was more often administered in extremity (57.3%) than in head (41.5%) or spine injuries (32.3%). An intravenous access was less often established in children < 6 years (74.3% vs. 90.8%; p < 0.001). Despite the use of potent analgesics, 396 missions (13.7%) were performed without technical monitoring. Particularly regarding patient data at handover in hospital, merely < 10% of all missions featured complete documentation. Therefore, sufficient evaluation of the efficacy of pain relief was not possible. Yet, by means of respiratory measures required during transport, severe side effects such as respiratory insufficiency, were barely noted.

**Conclusions:**

In the physician-staffed HEMS setting, pediatric trauma patients liberally receive opioids and Esketamine for analgesia. With regard to severe respiratory insufficiency during transport, the application of these potent analgesics seems safe.

## Introduction

Immediate pain relief in emergency critical care is more than an ethical obligation and of major importance to prevent adverse physiological and emotional side-effects [1–3]. Unfortunately, an under-use of analgetic drugs has been noted in emergency patients. Oligo-analgesia is typically driven by the fear of possible severe side effects of pain killers such as respiratory or circulatory depression or agitation [4, 5]. In this regard, children present a special challenge for most health care providers. First, children are rarely encountered in emergency medical services (EMS) [6]. Second, weight and pharmacodynamics and -kinetics differ substantially from adults [7]. Finally, a lack of practice and uncertainties in required dosages of potent analgesia might explain why pain in children is often insufficiently assessed and treated [8–11].

Helicopter emergency medical services (HEMS) typically respond to severely ill and injured children. Due to the nature of HEMS missions (i.e. alpine rescue flights, limited in-cabin treatment options) pain management is challenging. Safeguarding vital functions and handling of side effects is more demanding when compared with ground EMS. Thus, in the specific HEMS setting, insufficient analgesia in children might be an issue.

The aim of this study was to assess analgesia in injured, not mechanically ventilated children treated by HEMS over a 12-year timeframe in Austria. Indications, dosages and analgesic regimens were analyzed. Moreover, effectiveness and safety of drug administration in the HEMS setting was evaluated.

## Materials and methods

Retrospective study, approved by the Ethics Committee of the Medical University of Innsbruck (AN2015-0068 347/4.13 393/5.20), registered under the Clinical Trials number NCT03760302.

Data from 14 year-round helicopter bases in Austria, operated by the ÖAMTC Air Rescue (Austrian Automobile, Motorcycle and Touring Club) was analyzed in the timeframe from 01/01/2006 to 31/12/2017. The ÖAMTC HEMS crew consists of a pilot, an emergency medical technician (with advanced basic life support and mountain rescuer skills) and an emergency physician. The latter are typically advanced life support (ALS) certified and experienced in anesthesia and intensive care medicine (~ 80%). Rescue missions are documented with a standardized handwritten report form on-site, followed by a digital documentation after returning to the HEMS base.

Data obtained from this digital database included date, time, sex, age in groups, location of the helicopter base, type of HEMS operation (primary or secondary mission), emergency classifications, required medical disciplines, injury patterns, medications administered, and interventions performed by the emergency team. Emergency classifications mainly included *mountain accidents*, *other accidents* such as work, road traffic, home and leisure accidents, and *medical* emergencies, comprising non-traumatic pediatric, internal, psychiatric and neurological emergencies. Other rather infrequent emergencies (i.e. intoxications, obstetrics, suicides) were classified as *other*. Pain was graded by a Numeric Rating Scale (NRS) guided three tier scale including no pain, mild pain (NRS ≤ 3) and moderate to severe pain (NRS > 3). Regarding the analyzed pediatric patients, we must assume that pain levels were, at least partially, graded by physicians based on their own estimation. Within the registry, age is available as grouped variable only (5-to-10-year steps). A pediatric patient was defined to be < 15 years of age. Non-pediatric, non-primary missions were excluded (Fig. 3). Patients receiving any analgetic drugs were extracted and further analyzed after exclusion of patients mechanically ventilated on-site or uninjured. Patients without any documentation of an injured body part were considered uninjured.

The resulting age groups (0- to 5-, 6- to 10- and 11- to 14-year-olds) were further analyzed separately. The dosage and route of administration of analgetic drugs was assessed. Severity and progress of the patient’s condition was evaluated using the NACA (National Advisory Committee for Aeronautics) and MEES scoring (Mainz Emergency Evaluation Score) calculated by Glasgow Coma Scale, heart and respiratory rate, cardiac rhythm, pain, blood pressure and peripheral oxygen saturation (SpO_2_) [12, 13]. While NACA scores were assessed once on arrival on the scene, MEES scores were assessed on arrival and on handover in hospital.

Continuous data was tested for normal distribution via Shapiro–Wilk test. Due to non-normal distribution, particularly regarding NACA scores and analgetic dosages within the age groups, data are presented as median and interquartile range or count and percentage. The chi-square-test was performed to detect group differences in frequencies, the Kruskal–Wallis test for group differences of continuous data. Missing data were removed from analysis when comparing patient specific variables. Data were stored with Excel 2019 (Microsoft, Seattle, WA) and processed with R (v4.0.2, R Core Team, www.R-project.org) and RStudio (v1.2.5001, RStudio, Inc., Boston, MA).

## Results

### Demographics and general findings

In the 12-year time frame, HEMS responded to 176,056 patients including 14,425 (8.2%) children younger than 15 years (Fig. 3). Primary missions accounted for 12,324 (85.4%) pediatric cases. Of the 31.4% (n = 3874) children receiving analgesic drugs, non-trauma (n = 696) and on-site anesthetized children (n = 293) were excluded, thus resulting in 2885 patients for further analysis.

Based on the assumption that different age groups have specific needs, we identified 443 children (15.4%) under 6 years, 902 (31.3%) between 6 and 10 years, and 1540 (53.4%) between 11 and 14 years (Fig. 3). Emergency characteristics, pain levels, administered analgesia and additional interventions are shown in Table 1. Approximately two thirds were male and one third female. Median NACA score was 3 (3–4) regardless of age, and approximately 70% presented with one injured body region only.

**Table 1:** Emergency characteristics of children receiving medical analgesia during HEMS operations

	Injured, not mechanically ventilated children (< 15 yrs.) receiving medical analgesia (n = 2885) n (%) or median (IQR)			p
Age [yrs]	0–5 n = 443 (15.4)	6–10 n = 902 (31.3)	11–14 n = 1540 (53.4)	
Sex [male/female]	280/163 (63.2/36.8)	558/343 (61.9/38.1)	1048/492 (68.1/31.9)	
NA	0	1 (0.1)	0	
NACA score	3 (3–4)	3 (3–4)	3 (3–4)	< 0.001
NA	0	0	0	
*Number of injured regions*				
1	306 (69.1)	659 (73.1)	1085 (70.5)	0.237
2–3	125 (28.2)	221 (24.5)	420 (27.3)	0.225
≥ 4	12 (2.7)	22 (2.4)	35 (2.3)	0.864
NA	0	0	0	
*Injury localization*				
Head	144 (32.5)	193 (21.4)	290 (18.8)	< 0.001
Spine	16 (3.6)	116 (12.9)	252 (16.4)	< 0.001
Chest	81 (18.3)	74 (8.2)	153 (9.9)	< 0.001
Abdomen	50 (11.3)	109 (12.1)	165 (10.7)	0.585
Pelvis	10 (2.3)	22 (2.4)	55 (3.6)	0.172
Upper extremity	163 (36.8)	290 (32.2)	525 (34.1)	0.233
Lower extremity	186 (42.0)	451 (50.0)	755 (49.0)	0.015
*Initial level of pain*				
No pain	18 (4.1)	15 (1.7)	22 (1.4)	0.001
Mild pain	76 (17.4)	110 (12.4)	157 (10.3)	< 0.001
Moderate to severe pain	331 (75.7)	750 (84.4)	1338 (87.8)	< 0.001
NA	6 (1.4)	13 (1.4)	16 (1.0)	
*Analgesics administered*				
Opioids	165 (37.2)	490 (54.3)	977 (63.4)	< 0.001
Esketamine	293 (66.1)	487 (54.0)	744 (48.3)	< 0.001
Metamizole	5 (1.1)	18 (2.0)	38 (2.5)	0.216
NSAIDs	1 (0.2)	7 (0.8)	8 (0.5)	0.426
Acetaminophen	15 (3.4)	4 (0.4)	1 (0.1)	< 0.001
*Analgesic regimen*				
Mono therapy	408 (92.1)	798 (88.5)	1312 (85.2)	< 0.001
Dual therapy	34 (7.7)	104 (11.5)	226 (14.7)	< 0.001
Triple therapy	1 (0.2)	0 (0)	2 (0.1)	0.434
*Medical combinations*				
Opioids only	135 (30.5)	386 (42.8)	756 (49.1)	< 0.001
Esketamine only	259 (58.5)	392 (43.5)	536 (34.8)	< 0.001
Opioids with Esketamine	29 (6.5)	95 (10.5)	200 (13.0)	< 0.001
Metamizole only	2 (0.5)	13 (1.4)	18 (1.2)	0.274
Opioids with Metamizole	0 (0)	5 (0.6)	15 (1.0)	0.078
Acetaminophen only	12 (2.7)	2 (0.2)	0 (1.0)	< 0.001
*Route of administration*				
I.V. access	329 (74.3)	818 (90.7)	1399 (90.8)	< 0.001
I.O. access	4 (0.9)	0 (0.0)	1 (0.1)	< 0.001
Mucosal atomization device	8 (1.8)	13 (1.4)	6 (0.4)	0.004
NA	14 (3.2)	14 (1.6)	29 (1.9)	
*Technical monitoring applied*				
None	54 (12.2)	111 (12.3)	231 (15.0)	0.104
Blood pressure	168 (37.9)	432 (47.9)	823 (53.4)	< 0.001
Pulse oximetry	362 (81.7)	743 (81.2)	1165 (75.6)	0.001
3-channel-ECG	101 (22.8)	188 (20.8)	339 (22.0)	0.676
NA	0	0	0	

CPR denotes cardiopulmonary resuscitation, ECG electrocardiogram, I.O. intraosseus, IQR interquartile range, IV intravenous, NACA National Advisory Committee for Aeronautics, NA missing values

Children < 6 years suffered more frequently from head or chest injuries, while lower extremity and spine injuries were more frequent in the older age groups (Table 1). Intravenous lines where less often placed in the youngest age group (74.3% vs. 90.7% and 90.8%). In 396 HEMS operations (13.7%) vital signs monitoring was spared.

### Analgetic drugs administered

**Fig. 1:**
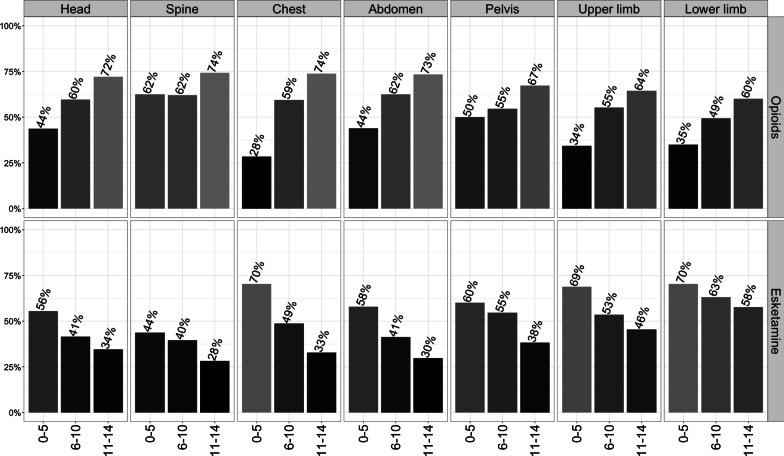
Proportional administration of potent analgesics (opioids, Esketamine) in dependency on injury location and age group. Shades of grey additionally indicate given proportions (darker < lighter)

In total, 2829 (98.1%) received an opioid and/or Esketamine. The analgesic concept typically chosen by HEMS physicians was a monotherapy with an opioid (n = 1277) or Esketamine (n = 1187), while the combination of both drugs (n = 324) was less frequently used (Table 1). Opioid use increased and Esketamine use decreased with age, regardless of injury localization (Fig. 1). Yet, Esketamine was more often administered in extremity (57.3%) than in head (41.5%) or spine injuries (32.3%). The most frequently administered opioid was Fentanyl followed by Piritramide and Morphine. The preferred use of Fentanyl vs. Piritramide was most evident in the youngest age group. Dosages predominantly increased adequately with age (Table 2). Merely Fentanyl dosages did not differ significantly between the two youngest age groups (p = 0.223). Despite also identical median dosages in the two youngest age groups, Esketamine dosages differed significantly due to differing interquartile ranges (p = 0.006).

**Table 2:** Dosages and frequencies of administration of potent analgesics

	n (%) or median (IQR)			p
Age [yrs]	0–5 n = 443	6–10 n = 902	11–14 n = 1540	
*Fentanyl*				
n total	117 (26.4)	268 (29.7)	496 (32.2)	0.053
Proportion with documented dosage	55 (47.0)	166 (61.9)	270 (48.0)	
Dosage [mg]	0.05 (0.03–0.07)	0.05 (0.05–0.10)	0.10 (0.05–0.20)	< 0.001
*Piritramide*				
n total	46 (10.4)	210 (23.3)	462 (30.0)	< 0.001
Proportion with documented dosage	26 (56.5)	140 (66.7)	299 (64.7)	
Dosage [mg]	2.38 (1.50–3.94)	4.00 (3.00–7.50)	7.50 (3.75–7.50)	< 0.001
*Morphine*				
n total	1 (0.2)	12 (0.1)	14 (0.9)	0.140
Proportion with documented dosage	1 (100.0)	6 (50.0)	10 (71.4)	
Dosage [mg]	1.5	4.00 (4.00–5.00)	5.00 (3.50–6.00)	0.251
*Esketamine*				
n total	293 (66.1)	487 (54.0)	744 (48.3)	< 0.001
Proportion with documented dosage	156 (53.2)	258 (53.0)	325 (43.7)	
Dosage [mg]	15.0 (10.0–25.0)	15.0 (12.5–25.0)	20.0 (15.0–25.0)	< 0.001

IQR denotes interquartile range

### Safety and efficacy of analgesics administered

**Fig. 2:**
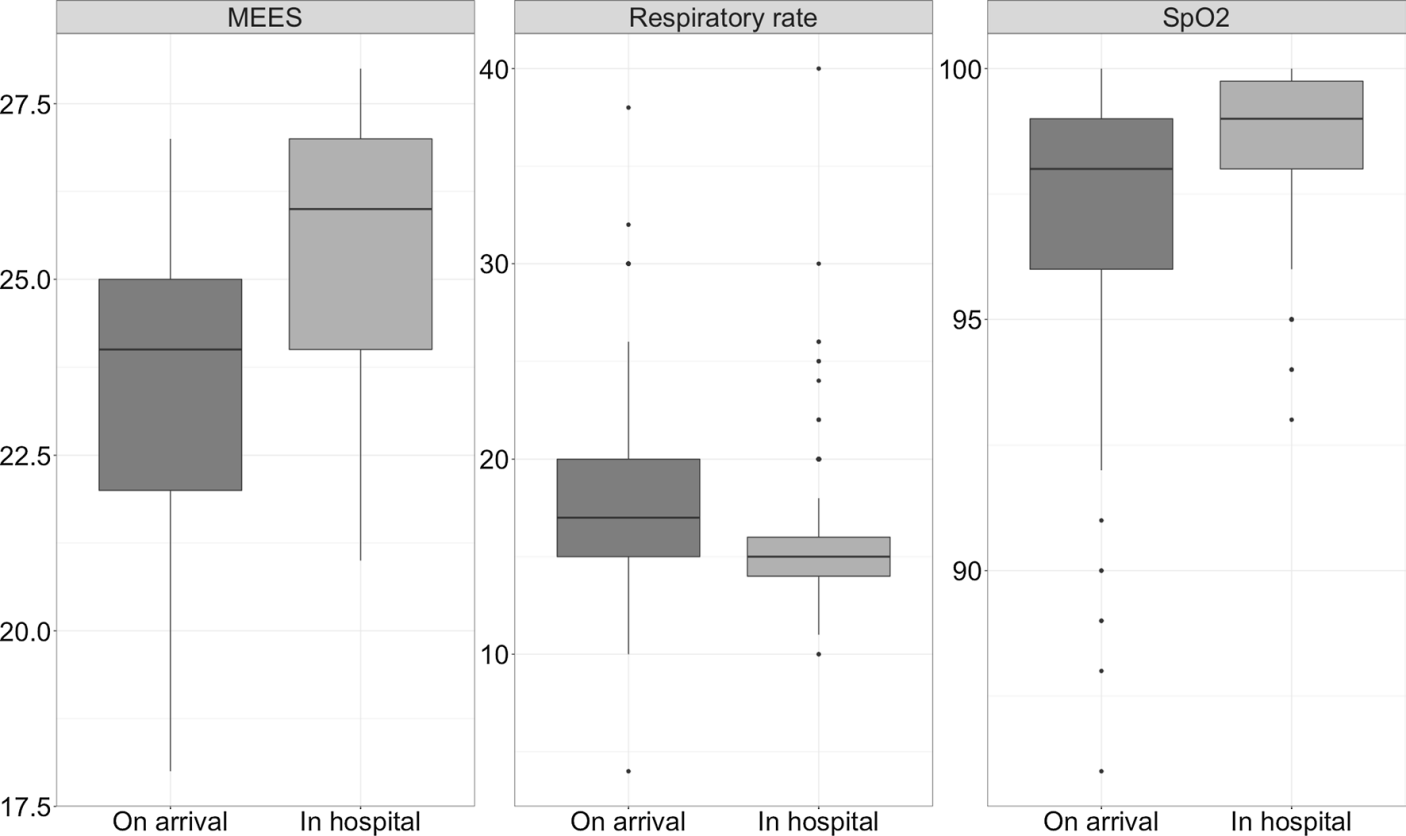
Development of MEES-scores, respiratory rates and SpO_2_ of children receiving opioids or Esketamine (n = 2829). On-site arrival of emergency physician compared to handover in hospital. Only patients with complete data with regard to the category of interest were included, resulting in n = 156 (5.5%) for total MEES-scores, n = 257 (9.1%) for respiratory rates and n = 262 (9.3%) for SpO_2_. Median NACA scores of patients with complete datasets: 4 (3–4) vs. 3 (3–4)

**Fig. 3:**
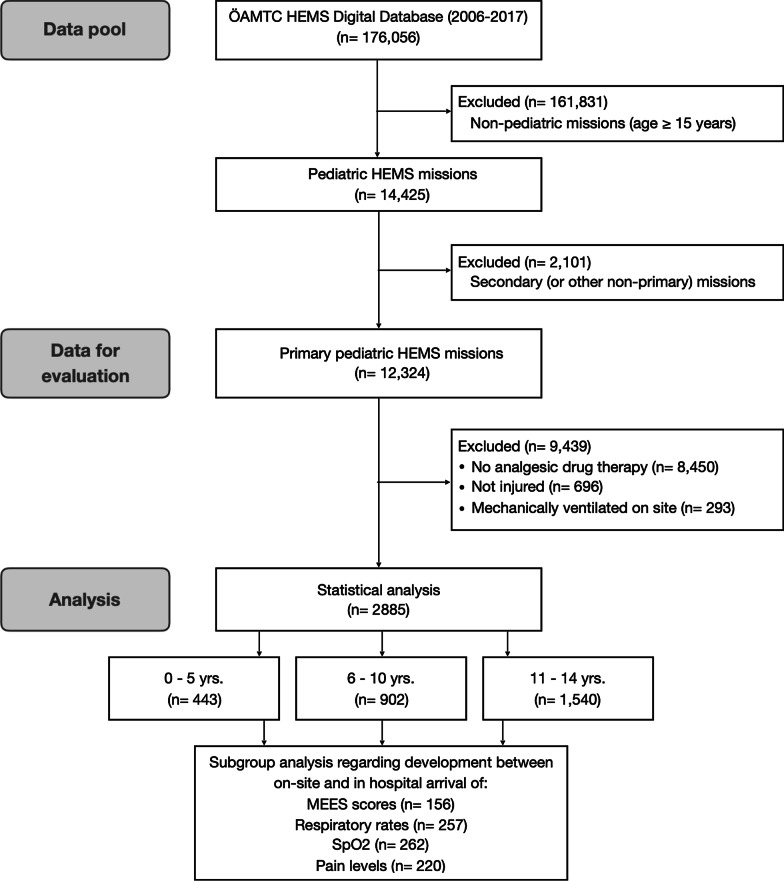
Consort flowchart

Administration safety was evaluated by the development of MEES-Scores, respiratory rates and SpO_2_ of patients receiving opioids or Esketamine (n = 2829). Measures between on-site arrival of the emergency physician and in hospital handover were compared. Unfortunately, merely < 10% of all missions featured complete documentation in this regard. Particularly documentation of patient data on handover in hospital were scarce (n = 156 (5.5%) for MEES-Scores, n = 257 (9.1%) for respiratory rates, n = 262 (9.3%) for SpO_2_). For the sake of completeness, data are shown in Fig. 2, but due to abovementioned reasons sincere conclusions cannot be drawn from these data alone. Importantly, NACA scores were significantly higher in patients with a complete dataset (4 (3–4) vs. 3 (3–4); p < 0.001) indicating a more severe condition and possibly a higher risk for side effects. Noteworthy, of the 2829 children receiving opioids or Esketamine, 1918 (67.8%) had an entry in respiratory measures required during transport. Hereof, 1216 (63.4%) required no additional measures, 691 (36.0%) oxygen supplementation and merely 2 (0.1%) tracheal intubation. The analyzed registry included 293 injured children receiving analgesics and requiring intubation on-site. Hereof, 78.8% were classified as NACA ≥ 5 (median 5; IQR 5–5), translating to acute danger, respiratory and/or cardiac arrest or death.

Efficacy of analgesics was evaluated by comparing pain levels between on-site arrival of the emergency physician and in hospital handover of patients receiving opioids or Esketamine. However, documentation quality was even lower in this regard (< 8% complete documentation). Therefore, we resigned from further analysis.

## Discussion

Our results, comprising data from 14 physician staffed HEMS in Austria during a 12-year time frame unveiled that opioids and Esketamine were the drugs of choice in injured children < 15 years requiring analgesia. The most common analgesic regimens were a mono therapy with opioids or Esketamine, while the combination of both was less frequently used. Severe respiratory insufficiency was hardly noted during transport, indicating safety of these two potent analgesic drugs.

### Demographics and general findings

Analgesia provided by HEMS in adults has been reported in military and civilian settings [5, 14–23]. Although there is evidence that pain treatment in children is insufficient [8–11], literature focusing on analgesia in children transported by HEMS are spare [24–26]. Since the HEMS setting differs significantly from ground EMS work, data may not be compared between both rescue systems. Further interfering with comparability are discrepancies in age thresholds when defining a patient to be pediatric. While these thresholds varied from – as in this study – 14 [26], to 15 [24, 25, 27, 28], 18 [6, 29, 30] or 20 years [8], some studies also excluded patients under 3 years of age [29]. The relative amount of pediatric emergencies encountered by EMS is typically low, accounting for 13–25% of all patients [6, 30]. In our study, we found only 8% of all missions dedicated to children with a high injury severity. As expected and shown in studies before, two thirds of the injured children were male [6, 24, 28].

The proportion of children receiving analgesics was 31%. Primarily depending on qualification and competencies of EMS personnel and inclusion criteria, this fraction has been reported from as low as 0.3% for a paramedic staffed EMS up to 92% for critical care physician staffed EMS units [9, 24, 30, 31]. Even when suffering from fractures, only some 10–37% of children are estimated to receive analgesics prehospitally [8, 32, 33]. Compared with a recent study from Australia including only severely injured children transported by ground or air-medical services to a pediatric trauma center IV line placement was about twofold higher in this study [28]. This is of special interest, as a lack of IV access has been shown to decrease frequency of analgesia in pediatric patients [24, 29]. Alternative administration routes such as intraosseous or intranasal application devices are still infrequently used [29].

### Analgetic drugs administered

The majority of children receiving analgesic drugs were injured, which is in line with previous studies describing proportions of 53–76% of all children requiring EMS to be injured [6, 34, 35]. The most common analgesic regimens were a mono therapy with opioids or Esketamine followed by a combination therapy of opioids and Esketamine. Fentanyl was the most commonly administered opioid with its preference also described by Johnson et al. [24]. With increasing age, opioids were used more frequently, while the use of Esketamine decreased. Although this trend was independent of injury localization, Esketamine was more often administered in extremity than in head or spine injuries. Controversies about the use of Ketamine in head injuries still prevail [36, 37]. The liberal use of the racemate Ketamine or its enantiomer Esketamine in an EMS setting, particularly with regard to its use in children is a priori dependent on national and regional regulations. In Austria, Esketamine is predominantly used over the racemate Ketamine since 10 + years due to its better side-effect profile [38]. Similar to the data presented here, a study from London, UK, stated that Ketamine was the preferred medication for on-scene pediatric analgesia [36].

With regard to opioids, many studies have shown their insufficient use in children in EMS settings. Fractions range from 2 to 32% of injured children to receive opioids [8, 9, 29, 32, 39–41] or 15% of severely injured children in HEMS to receive Ketamine or Fentanyl [25]. In our study, nearly all (> 95%) children who received analgesia, received an opioid and/or Esketamine. This high proportion of potent analgesics among injured children receiving any kind of analgesia stands in strong contrast to other studies describing opioid use in merely 13% of children receiving analgesia for fractures [32]. A decreased fraction of analgesia, in particular of opioid use in the youngest children (< 5 years), as seen in the presented data, has been described before [24, 27, 39, 41] and has led to numerous pleas demanding that also neonates and young infants should receive adequate pain relief [7, 11].

### Safety and efficacy of analgesics administered

The inadequate treatment of children suffering severe pain is under discussion [1, 42] and studies continue to demonstrate an underuse of analgesic agents, thus resulting in oligo-analgesia among pediatric patients [7–9]. In this regard, appropriate pain assessment and documentation is acknowledged to be of utmost important [8, 11, 43] to trigger and monitor pre-hospital analgesia. Nevertheless, poor documentation of pain scores is rather common, especially among pediatric patients where documented pain is described in as few as 4% [24, 29, 44–47]. In our study, we found the pain NRS score documented in 99% of all patients receiving analgesic drugs at the time of first contact, but only in 8% before or during handover in hospital. Therefore, sincere analysis of pain relief was deemed unsound.

Potent central analgesics, especially opioids, are feared for their potentially severe adverse events – in particular respiratory depression. In the specific HEMS setting in-cabin space and patient access is limited, thus hampering the options to interfere and treat respiratory deterioration during flight. Literature on application safety of prehospitally administered analgesics is growing both for Esketamine and opioids. The afore mentioned study from London, UK, described liberal racemate Ketamine use (mean 1 mg/kg), predominantly in awake non-trapped children with blunt trauma [36] and did not demonstrate any major side effects, especially with regard to loss of airway patency. These findings were in line with another small study (n = 40) reporting no adverse events of Ketamine during air transport [48]. Concerning opioids, two studies showed no substantial respiratory depression, hypotension or other clinically significant adverse effect attributable to Fentanyl (1–3 µg/kg) in pediatric air-transported trauma patients [26, 44]. Especially the use of pain protocols addressing pain medication were shown to safely and effectively increase the frequency of analgesia without causing any major side effects [24, 44]. It is noteworthy that pediatric analgesia in our study was not driven by elaborated protocols. Moreover, HEMS in Austria is typically physician staffed and the vast majority of HEMS physicians (80%) are trained anesthesiologists with specific training in prehospital emergency medicine. The development of MEES-Scores, respiratory rates and SpO_2_ values in children not requiring mechanical ventilation on-site, indicated application safety but data was too incomplete to draw sincere conclusions. However, more than two-thirds of children receiving opioids or Esketamine had an entry in respiratory measures required during transport. Hereof, nearly two-thirds did not require any additional measures at all, and more than one-third merely received oxygen supplementation during transport. Of course, severe side effects causing immediate intubation on-site may have been missed in our analysis as on-site intubated patients were primarily excluded. Additional analysis however revealed, that from the 293 injured children receiving analgesic medication and requiring intubation on-site, 78.8% were classified as NACA ≥ 5. Therefore, we truly believe that intubation was indicated by injury severity rather than medication side effects. In accordance with existent literature, we assume application safety of potent analgesics, when administered by protocol or by an experienced physician.

### Limitations

Complete documentation, including both values from initial on-site evaluation and later arrival in hospital was present only in 156 cases regarding MEES-Scores (5.5%), 257 cases regarding SpO_2_ values (9.1%), 262 cases regarding respiratory rates (9.3%) and n = 220 cases regarding pain levels (7.9%). As mentioned before, poor documentation quality is not uncommon in emergency settings and reporting bias can therefore not be excluded. Furthermore, pain levels were documented by utilizing an NRS guided scale but not by exact numeric documentation. As children may often not be capable of adequately assessing an NRS, we must assume that pain-levels were often approximated by the HEMS-physician. As a result, detailed analysis of pain reduction was therefore not possible. Also due to incomplete documentation, weight dependent dosages of administered analgesia could not be specified. Furthermore, data analysis in general was conducted retrospectively.

## Conclusions

In this physician-staffed HEMS setting, pediatric trauma patients liberally receive opioids and Esketamine for analgesia. With regard to severe respiratory insufficiency during transport, the application of these potent analgesics seems safe.

## Data Availability

No data are available. Participant data from the ÖAMTC CFV. All data are deidentified. The data set was delivered containing only serial numbers for each participant. Protocol and statistical analysis plans are available.

## Appendix

See Fig. 3.

## Declarations

### Patient and public involvement

Patients and/or the public were not involved in the design, conduct of this study, or reporting of this research.

## References

[1] Howard RF (2003). Current status of pain management in children. JAMA.

[2] Weisman SJ, Bernstein B, Schechter NL (1998). Consequences of inadequate analgesia during painful procedures in children. Arch Pediatr Adolesc Med.

[3] McManus JG, Sallee DR (2005). Pain management in the prehospital environment. Emerg Med Clin North Am.

[4] Kiavialaitis G, Müller S, Braun J, Rössler J, Spahn D, Stein P, Kaserer A (2019). Clinical practice of pre-hospital analgesia: an observational study of 20,978 missions in Switzerland. Am J Emerg Med.

[5] Scholten AC, Berben SAA, Westmaas AH, van Grunsven PM, de Vaal ET, Rood PPM, Hoogerwerf N, Doggen CJM, Schoonhoven L (2015). Pain management in trauma patients in (pre)hospital based emergency care: current practice versus new guideline. Injury.

[6] Shah MN, Cushman JT, Davis CO, Bazarian JJ, Auinger P, Friedman B (2009). The epidemiology of emergency medical services use by children: an analysis of the National Hospital Ambulatory Medical Care Survey. Prehosp Emerg Care.

[7] Williams DM, Rindal KE, Cushman JT, Shah MN (2012). Barriers to and enablers for prehospital analgesia for pediatric patients. Prehosp Emerg Care.

[8] Swor R, McEachin CM, Seguin D, Grall KH (2009). Prehospital pain management in children suffering traumatic injury. Prehosp Emerg Care.

[9] Izsak E, Moore JL, Stringfellow K, Oswanski MF, Lindstrom DA, Stombaugh HA (2009). Prehospital pain assessment in pediatric trauma. Prehosp Emerg Care.

[10] Bendall JC, Simpson PM, Middleton PM (2011). Prehospital analgesia in New South Wales, Australia. Prehospital Disaster Med.

[11] Fein JA, Zempsky WT, Cravero JP, Medicine C on PEM and S on A and P, Pediatrics AA. Relief of pain and anxiety in pediatric patients in emergency medical systems. Pediatrics. 2012;130:e1391–e1405. 10.1542/peds.2012-2536. 10.1542/peds.2012-2536 23109683

[12] Alessandrini H, Oberladstätter D, Trimmel H, Jahn B, Baubin M (2012). NACA-Scoringsystem: Eine retro- und prospektive Validitätsanalyse anhand ausgewählter Diagnosegruppen. Notf Rettungsmedizin.

[13] Reinhardt T, Hennes H-J (1999). Mainz emergency evaluation score (MEES). Notf Rettungsmedizin.

[14] Helm M, Hossfeld B, Braun B, Werner D, Peter L, Kulla M (2020). Oligoanalgesia in patients with an initial Glasgow coma scale score ≥8 in a physician-staffed helicopter emergency medical service: a multicentric secondary data analysis of >100,000 out-of-hospital emergency missions. Anesth Analg.

[15] Albrecht E, Taffe P, Yersin B, Schoettker P, Decosterd I, Hugli O (2013). Undertreatment of acute pain (oligoanalgesia) and medical practice variation in prehospital analgesia of adult trauma patients: a 10 yr retrospective study. Br J Anaesth.

[16] Oberholzer N, Kaserer A, Albrecht R, Seifert B, Tissi M, Spahn DR, Maurer K, Stein P (2017). Factors influencing quality of pain management in a physician staffed helicopter emergency medical service. Anesth Analg.

[17] Mora AG, Ganem VJ, Ervin AT, Maddry JK, Bebarta VS (2016). En route use of analgesics in nonintubated, critically ill patients transported by U.S. Air Force Critical Care Air Transport Teams. Mil Med.

[18] Shackelford SA, Fowler M, Schultz K, Summers A, Galvagno SM, Gross KR, Mabry RL, Bailey JA, Kotwal RS, Butler FK (2015). Prehospital pain medication use by U.S. Forces in Afghanistan. Mil Med.

[19] Schauer SG, Mora AG, Maddry JK, Bebarta VS (2017). Multicenter, prospective study of prehospital administration of analgesia in the U.S. Combat Theater of Afghanistan. Prehospital Emerg Care Off J Natl Assoc EMS Physicians Natl Assoc State EMS Dir.

[20] Petz LN, Tyner S, Barnard E, Ervin A, Mora A, Clifford J, Fowler M, Bebarta VS (2015). Prehospital and en route analgesic use in the combat setting: a prospectively designed, multicentre, observational study. Mil Med.

[21] Pasquier M, Geiser V, De Riedmatten M, Carron PN (2012). Helicopter rescue operations involving winching of an emergency physician. Injury.

[22] Samdal M, Haugland HH, Fjeldet C, Rehn M, Sandberg M (2018). Static rope evacuation by helicopter emergency medical services in rescue operations in southeast Norway. Wilderness Environ Med.

[23] Rugg C, Woyke S, Voelckel W, Paal P, Ströhle M (2021). Analgesia in adult trauma patients in physician-staffed Austrian helicopter rescue: a 12-year registry analysis. Scand J Trauma Resusc Emerg Med.

[24] Johnson TJ, Schultz BR, Guyette FX (2014). Characterizing analgesic use during air medical transport of injured children. Prehosp Emerg Care.

[25] Barker CL, Weatherall AD (2014). Prehospital paediatric emergencies treated by an Australian Helicopter Emergency Medical Service. Eur J Emerg Med Off J Eur Soc Emerg Med.

[26] DeVellis P, Thomas SH, Wedel SK, Stein JP, Vinci RJ (1998). Prehospital fentanyl analgesia in airtransported pediatric trauma patients. Pediatr Emerg Care.

[27] Watkins N (2006). Paediatric prehospital analgesia in Auckland. Emerg Med Australas.

[28] Curtis K, Kennedy B, Lam MK, Mitchell RJ, Black D, Burns B, Loudfoot A, Tall G, Dinh M, Beech C (2020). Prehospital care and transport costs of severely injured children in NSW Australia. Injury.

[29] Browne LR, Studnek JR, Shah MI, Brousseau DC, Guse CE, Lerner EB (2016). Prehospital opioid administration in the emergency care of injured children. Prehospital Emerg Care Off J Natl Assoc EMS Physicians Natl Assoc State EMS Dir.

[30] Lerner EB, Dayan PS, Brown K, Fuchs S, Leonard J, Borgialli D, Babcock L, Hoyle JD, Kwok M, Lillis K (2013). Characteristics of the pediatric patients treated by the pediatric emergency care applied research network’s affiliated EMS agencies. Prehosp Emerg Care.

[31] Galinski M, Picco N, Hennequin B, Raphael V, Ayachi A, Beruben A, Lapostolle F, Adnet F (2011). Out-of-Hospital emergency medicine in pediatric patients: prevalence and management of pain. Am J Emerg Med.

[32] Rogovik AL, Goldman RD (2007). Prehospital use of analgesics at home or en route to the hospital in children with extremity injuries. Am J Emerg Med.

[33] Dong L, Donaldson A, Metzger R, Keenan H (2012). Analgesic administration in the emergency department for children requiring hospitalization for long-bone fracture. Pediatr Emerg Care.

[34] McEachin CC, McDermott JT, Swor R (2009). Few emergency medical services patients with lower-extremity fractures receive prehospital analgesia. Prehosp Emerg Care.

[35] Singer AJ, Gulla J, Thode HC (2002). Parents and practitioners are poor judges of young children’s pain severity. Acad Emerg Med.

[36] Bredmose PP, Lockey DJ, Grier G, Watts B, Davies G (2009). Pre-hospital use of ketamine for analgesia and procedural sedation. Emerg Med J EMJ.

[37] Gregers MCT, Mikkelsen S, Lindvig KP, Brøchner AC (2020). Ketamine as an anesthetic for patients with acute brain injury: a systematic review. Neurocrit Care.

[38] Marland S, Ellerton J, Andolfatto G, Strapazzon G, Thomassen O, Brandner B, Weatherall A, Paal P (2013). Ketamine: use in anesthesia. CNS Neurosci Ther.

[39] White LJ, Cooper JD, Chambers RM, Gradisek RE (2000). Prehospital use of analgesia for suspected extremity fractures. Prehosp Emerg Care.

[40] Samuel N, Steiner IP, Shavit I (2015). Prehospital pain management of injured children: a systematic review of current evidence. Am J Emerg Med.

[41] Rutkowska A, Skotnicka-Klonowicz G (2015). Prehospital pain management in children with traumatic injuries. Pediatr Emerg Care.

[42] Schechter NL, Allen DA, Hanson K (1986). Status of pediatric pain control: a comparison of hospital analgesic usage in children and adults. Pediatrics.

[43] Whitley GA, Hemingway P, Law GR, Jones AW, Curtis F, Siriwardena AN (2020). The predictors, barriers and facilitators to effective management of acute pain in children by emergency medical services: a systematic mixed studies review. J Child Health Care.

[44] Thomas SH, Rago O, Harrison T, Biddinger PD, Wedel SK (2005). Fentanyl trauma analgesia use in air medical scene transports. J Emerg Med.

[45] Drendel AL, Brousseau DC, Gorelick MH (2006). Pain assessment for pediatric patients in the emergency department. Pediatrics.

[46] Rahman A, Curtis S, DeBruyne B, Sookram S, Thomson D, Lutz S, Ali S (2015). Emergency medical services provider comfort with prehospital analgesia administration to children. Prehospital Disaster Med.

[47] Hennes H, Kim MK, Pirrallo RG (2009). Prehospital pain management. Prehosp Emerg Care.

[48] Svenson JE, Abernathy MK (2007). Ketamine for prehospital use: new look at an old drug. Am J Emerg Med.

